# Exact explosive synchronization transitions in Kuramoto oscillators with time-delayed coupling

**DOI:** 10.1038/s41598-018-33845-6

**Published:** 2018-10-19

**Authors:** Hui Wu, Ling Kang, Zonghua Liu, Mukesh Dhamala

**Affiliations:** 1 Department of Mathematical Sciences, Clark Atlanta University, Atlanta, GA 30314 USA; 2 Department of Physics, East China Normal University, Shanghai, 200062 China; 3 Department of Physics and Astronomy, Neuroscience Institute, Center for Behavioral Neuroscience, Georgia State and Georgia Tech Center for Advanced Brain Imaging, Georgia State University, Atlanta, GA 30303 USA

## Abstract

Synchronization commonly occurs in many natural and man-made systems, from neurons in the brain to cardiac cells to power grids to Josephson junction arrays. Transitions to or out of synchrony for coupled oscillators depend on several factors, such as individual frequencies, coupling, interaction time delays and network structure-function relation. Here, using a generalized Kuramoto model of time-delay coupled phase oscillators with frequency-weighted coupling, we study the stability of incoherent and coherent states and the transitions to or out of explosive (abrupt, first-order like) phase synchronization. We analytically derive the exact formulas for the critical coupling strengths at different time delays in both directions of increasing (forward) and decreasing (backward) coupling strengths. We find that time-delay does not affect the transition for the backward direction but can shift the transition for the forward direction of increasing coupling strength. These results provide valuable insights into our understanding of dynamical mechanisms for explosive synchronization in presence of often unavoidable time delays present in many physical and biological systems.

## Introduction

In many physical, biological and technological oscillatory systems, useful function emerges from collective synchronization of an ensemble of constituent oscillators. Examples include working of neurons in the brain^1–3^, phase-locking of Josephson junction arrays^4,5^, the dynamics of power grids^6^. The Kuramoto model^7,8^, originally formulated to simplify the Winfree’s coupled oscillator model for the circadian rhythms of plants and animals^9^, remarkably generalizes to explain phase synchronization phenomena in these examples and many more^10,11^. Transitions to or out of synchronization as a consequence of changing coupling strength are analogous to phase transitions studied in statistical physics such as ferromagnetic, superconductive and thermodynamic transitions^12^.

Collective synchronization of coupled phase oscillators depends on several factors: intrinsic frequency distribution, coupling strength, interaction time-delays, network and structure (coupling strength or topology)- dynamics (frequency) relation. As the coupling strength is changed across a certain critical value, the transition from incoherent to coherent, or coherent to incoherent states takes place smoothly (the second-order phase transition like) or abruptly (the first-order like). When coupling is associated with oscillator characteristics or outputs, abrupt transitions to synchrony can occur with hysteresis in a variety of coupled oscillator systems, including in Josephon junction arrays^13^, in complex networks of oscillators^12,14–18^, and in frequency-weighted, mean-field coupled system of Kuramoto models^19^. Time-delay in mean-field coupling can also make the synchronization transition abrupt^20,21^. Despite our current advanced understanding of synchronization transitions in a variety of these systems, the effects of time-delay and frequency correlated mean-field coupling (structure-dynamics relation) in phase synchronization remain to be explored.

Interaction time-delays and structure-function interdependence are usually unavoidable characteristics of spatially distributed, adaptive oscillatory systems, such as neurons in the brain that has a functional organization^3,22,23^. In such systems, smooth or abrupt synchronization transitions may help us to distinguish between normal and abnormal functioning, such as pconerceptual decision-making^24^ as an example of normal functions and epileptic seizure as dysfunction^18,25^.

In this work, we analyze a generalized Kuramoto model of time-delay coupled phase oscillators with frequency-weighted global coupling for stability of incoherent states and coherent states and derive the exact analytical solutions for the critical coupling strengths at different time delays in both directions of increasing (forward) and decreasing (backward) coupling strengths. Here, as a general result, we will come to show that the time delay coupling can affect the abrupt synchronization transition only in the forward direction and not in the backward direction in various network topologies and distributed time-delays.

## Methods and Results

We consider the following generalized Kuramoto model with time-delay and frequency-weighted coupling:

1 $${\dot{\theta }}_{i}(t)={\omega }_{i}+\frac{\kappa }{N}|{\omega }_{i}|\,\sum _{j=1}^{N}\,\sin ({\theta }_{j}(t-\tau )-{\theta }_{i}(t))$$

Here, the coupled system consists of N number of oscillators, each with *θ*_*i*_(*t*) as the instantaneous phase at time *t*, ${\dot{\theta }}_{i}(t)$ its derivative and *ω*_*i*_ as the natural frequency. A set of N natural frequencies is drawn from a zero-centered symmetric distribution function (*g*(*ω*) = *g*(−*ω*)). The coupling strength *k* is modulated by |*ω*_*i*_|. The heterogeneity of couplings thus achieved can represent characteristics of adaptive oscillator systems commonly found in nature. The heterogeneity of couplings thus achieved can represent characteristics of many functionally organized and spatially distributed oscillator systems, some relevant examples of which include power grid networks^12,26^, social communication networks^27,28^ and brain neuronal oscillatory networks^3,29^.

Here, we consider the case of fully connected networks so that we can use the mean-field approach and the continuity equation for the time-evolution of instantaneous phase distribution *ρ*(*θ*, *ω*, *t*) on a unit circle. We define an order parameter *r* for *t* − *τ* time by

2 $$r{e}^{i\varphi (t-\tau )}=\frac{1}{N}\,\sum _{j=1}^{N}\,{e}^{i{\theta }_{j}(t-\tau )}$$

where *r* characterizes phase coherence and *ϕ* the average phase of the coupled system. In stationary state, the definition of Eq. (2) will be equivalent to the traditional definition of $r{e}^{i\varphi }=\frac{1}{N}\,{\sum }_{j=1}^{N}\,{e}^{i{\theta }_{j}}$ with no time-delay. Multiplying a factor ${e}^{-i{\theta }_{i}(t)}$ to both sides of Eq. (2) we have

3 $$r{e}^{i(\varphi (t-\tau )-{\theta }_{i}(t))}=\frac{1}{N}\,\sum _{j=1}^{N}\,{e}^{i({\theta }_{j}(t-\tau )-{\theta }_{i}(t))}$$

The imaginary part of Eq. (3) is:

4 $$r\,\sin (\varphi (t-\tau )-{\theta }_{i}(t))=\frac{1}{N}\,\sum _{j=1}^{N}\,\sin ({\theta }_{j}(t-\tau )-{\theta }_{i}(t))$$

With Eqs (1) and (4), we obtain:

5 $${\dot{\theta }}_{i}={\omega }_{i}+\kappa r|{\omega }_{i}|\,\sin (\varphi (t-\tau )-{\theta }_{i}(t))$$

Dependent on the coupling strength *κ* and time delay *τ* for a given frequency distribution *g*(*ω*), the coupled system as represented in Eq. (1), can show phase coherence (*r* > 0), or incoherence (*r* ≈ 0). For the forward (incoherent to coherent state) transition, we linearize the continuity equation around the incoherent state (*ρ*(*θ*, *ω*, *t*) = 1/2*π*) and obtain the critical coupling strength *K*_*f*_ for the forward direction. For the backward (coherent to incoherent state) transition, we start with fully coherent state (*r* = 1) at sufficiently large *κ* and use the self-consistency approach on the main mean-field equation to obtain the critical coupling strength *K*_*b*_ in the backward direction. Here, we show our calculations for a zero-centered Lorentzian distribution of frequencies, but the calculation method can be applied to any smooth symmetric frequency distribution.

### Forward phase transition

In the continuum limit $N\to \infty$, the probability density function (*ρ*(*θ*, *ω*, *t*)) that represents the fraction of oscillators with frequency *ω* whose phases are distributed between *θ* and *θ* + *dθ* satisfies (i) the normalizing condition: ${\int }_{0}^{2\pi }\,\rho (\theta ,\omega ,t)=1$ and (ii) the incoherent state value *ρ*_0_(*θ*, *ω*, *t*) = 1/2*π* uniformly distributed over the unit circle. We introduce a small perturbation to a completely incoherent state: ${\rho }_{0}(\theta ,\omega ,t)=\frac{1}{2\pi }$ with $\varepsilon \ll 1$:

6 $$\rho (\theta ,\omega ,t)=\frac{1}{2\pi }+\varepsilon \eta (\theta ,\omega ,t)$$

Since  ${\int }_{0}^{2\pi }\,\eta (\theta ,\omega ,t)d\theta =0$, we have

7 $$\begin{array}{ccc}r{e}^{i\varphi } & = & \frac{1}{N}\,\sum _{j=1}^{N}\,{e}^{i{\theta }_{j}}={\int }_{0}^{2\pi }\,{\int }_{-{\rm{\infty }}}^{{\rm{\infty }}}\,{e}^{i\theta }\rho (\theta ,\omega ,t)g(\omega )d\omega d\theta \\  & = & \varepsilon \,{\int }_{0}^{2\pi }\,{\int }_{-{\rm{\infty }}}^{{\rm{\infty }}}\,{e}^{i\theta }\eta (\theta ,\omega ,t)g(\omega )d\omega d\theta =\varepsilon {r}^{{\rm{^{\prime} }}}{e}^{i\varphi }\end{array}$$

We now get *r* = *εr*′ and

8 $$r{\rm{^{\prime} }}{e}^{i\varphi }={\int }_{0}^{2\pi }\,{\int }_{-{\rm{\infty }}}^{{\rm{\infty }}}\,{e}^{i\theta }\eta (\theta ,\omega ,t)g(\omega )d\omega d\theta .$$

The continuity equation for *ρ* is:

9 $$\frac{\partial \rho }{\partial t}+\frac{\partial (\rho v)}{\partial \theta }=0$$

The flow velocity function *v*(*t*) is

10 $$v(t)=\omega (t)+\varepsilon \kappa r{\rm{^{\prime} }}|\omega (t)|\,\sin (\varphi (t-\tau )-\theta (t))$$

By sustituting (6), (8), (10) into (9) and considering the consistency of *O*(*ε*) terms, we have

11 $$\frac{{\rm{\partial }}\eta }{{\rm{\partial }}t}=-\,\omega \frac{{\rm{\partial }}\eta }{{\rm{\partial }}\theta }+\frac{\kappa r{\rm{^{\prime} }}|\omega |\,\cos (\varphi (t-\tau )-\theta (t))}{2\pi }$$

Here *η*(*θ*, *ω*, *t*) can be expanded into the following complex Fourier series:

12 $$\eta (\theta ,\omega ,t)=c(\omega ,t){e}^{i\theta }+{c}^{\ast }(\omega ,t){e}^{-i\theta }+{\eta }^{\perp }(\theta ,\omega ,t)$$

${\eta }^{\perp }$ represents higher Fourier harmonics terms. Now,

13 $$\begin{array}{ccc}r{\rm{^{\prime} }}{e}^{i(\varphi (t-\tau )-\theta (t))} & = & {e}^{-i\theta (t)}r{\rm{^{\prime} }}{e}^{i\varphi (t-\tau )}\\  & = & {e}^{-i\theta (t)}\,{\int }_{0}^{2\pi }\,{\int }_{-{\rm{\infty }}}^{{\rm{\infty }}}\,{e}^{ix}\eta (x,\omega ,t-\tau )g(\omega )d\omega dx\\  & = & 2\pi {e}^{-i\theta }\,{\int }_{-{\rm{\infty }}}^{{\rm{\infty }}}\,{c}^{\ast }(\omega ,t-\tau )g(\omega )d\omega \end{array}$$

Similarly,

14 $$r{\rm{^{\prime} }}{e}^{-i(\varphi (t-\tau )-\theta (t))}=2\pi {e}^{i\theta }\,{\int }_{-{\rm{\infty }}}^{{\rm{\infty }}}\,c(\omega ,t-\tau )g(\omega )d\omega $$

Now, with Eqs (13) and (14),

15 $$\begin{array}{ccc}r{\rm{^{\prime} }}\,\cos (\varphi (t-\tau )-\theta (t)) & = & \pi [{e}^{-i\theta }\,{\int }_{-{\rm{\infty }}}^{{\rm{\infty }}}\,{c}^{\ast }(\omega ,t-\tau )g(\omega )d\omega \\  &  & +\,{e}^{i\theta }\,{\int }_{-{\rm{\infty }}}^{{\rm{\infty }}}\,c(\omega ,t-\tau )g(\omega )d\omega ]\end{array}$$

Using (12), (15) into (11) and comparing the coefficients of *e*^*iθ*^:

16 $$\frac{\partial c(\omega ,t)}{\partial t}=-\,i\omega c(\omega ,t)+\frac{\kappa |\omega |}{2}\,{\int }_{-\infty }^{\infty }\,c(v,t-\tau )g(v)dv$$

We now look for a separable solution: *c*(*ω*, *t*) = *b*(*ω*)*e*^*λt*^ in the equation (16):

17 $$\lambda b(\omega )=-\,i\omega b(\omega )+\frac{\kappa |\omega |{e}^{-\lambda \tau }}{2}\,{\int }_{-\infty }^{\infty }\,b(v)g(v)dv$$

Suppose $\frac{\kappa }{2}\,{\int }_{-\infty }^{\infty }\,b(v)g(v)dv=A$. Now, from (17), we have $b(\omega )={e}^{-\lambda \tau }\frac{|\omega |A}{\lambda +i\omega }$ and $\frac{\kappa {e}^{-\lambda \tau }}{2}\,{\int }_{-\infty }^{\infty }\,\frac{|\omega |A}{\lambda +i\omega }g(\omega )d\omega =A$. This leads to:

18 $${e}^{\lambda \tau }=\frac{\kappa }{2}\,{\int }_{-\infty }^{\infty }\,\frac{\lambda |\omega |}{{\lambda }^{2}+{\omega }^{2}}g(\omega )d\omega $$

With $g(\omega )=\frac{1}{\pi ({\omega }^{2}+1)}$ for a standard Lorentzian distribution, we have:

$${e}^{\lambda \tau }=\tfrac{\kappa }{\pi }\,{\int }_{0}^{+\infty }\,\tfrac{\lambda \omega }{{\lambda }^{2}+{\omega }^{2}}\cdot \tfrac{1}{{\omega }^{2}+1}d\omega =\tfrac{\kappa \lambda }{\pi (1-{\lambda }^{2})}\,{\int }_{0}^{+\infty }\,(\tfrac{\omega }{{\lambda }^{2}+{\omega }^{2}}-\tfrac{\omega }{{\omega }^{2}+1})\,d\omega =\tfrac{\kappa \lambda }{\pi (1-{\lambda }^{2})}\,(\,-\,\mathrm{ln}\,\lambda )=\tfrac{\kappa \lambda }{\pi ({\lambda }^{2}-1)}\,(\mathrm{ln}\,\lambda ).$$

Hence,

19 $$\kappa =\frac{\pi ({\lambda }^{2}-1){e}^{\lambda \tau }}{\lambda \,\mathrm{ln}\,\lambda }$$

As *R* = |*λ*| > 0, *κ* passes through the bifurcation point when *λ* = *iR* or *λ* = −*iR*. When *λ* = *iR*,

20 $${\kappa }_{1}=\frac{\pi (\,-\,{R}^{2}-1){e}^{iR\tau }}{iR(\mathrm{ln}\,R+\frac{\pi }{2}i)}$$

When *λ* = −*iR*,

21 $${\kappa }_{2}=\frac{\pi (\,-\,{R}^{2}-1){e}^{-iR\tau }}{-\,iR(\mathrm{ln}\,R-\frac{\pi }{2}i)}$$

We set ${\kappa }_{1}={\bar{\kappa }}_{2}$ (complex conjugate pairs) to seek for real critical values. When *τ* and *R* satisfies:

22 $$\tan (\tau R)=-\,\frac{2}{\pi }\,\mathrm{ln}\,R$$

*κ*_1_ = *κ*_2_ and both are real. Equation (22) has many number of intersection points. Only one *R* = *R*_0_ > 0 is a unique efficient solution and all others are extra roots. The forward critical value of *κ* is determined by the value of *R*_0_ as follows:

23 $${K}_{f}=\frac{2({R}_{0}^{2}+1)}{{R}_{0}}\,\cos (\tau {R}_{0})$$

**Figure 1:**
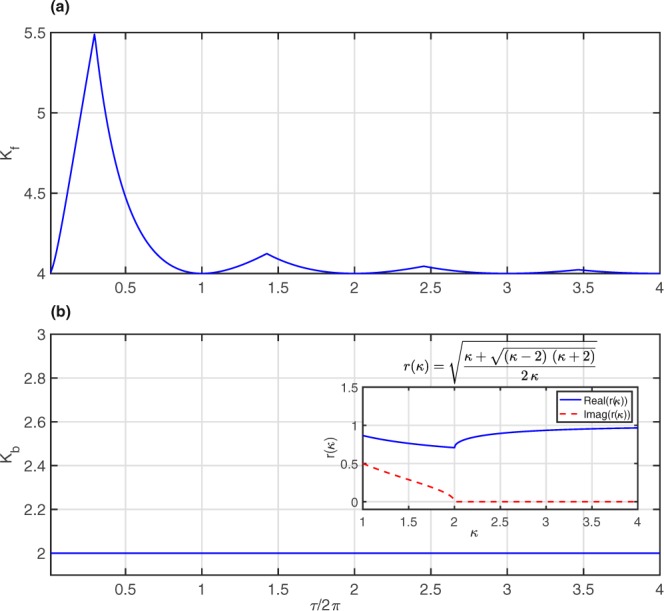
(**a**,**b**) Theoretical predictions of critical coupling strengths (*K*_*critical*_ = {*K*_*f*_, *K*_*b*_}) as a function of time delay (*τ*) for increasing (forward) and decreasing (backward) directions. The inset in (**b**) shows how the real (blue line) and imaginary (red dashed line) values of *r*(*κ*) vary with *κ* in the range $[14]$, which helps to determine the critical coupling to be *K*_*b*_ = 2 at *r*(*κ*) ≈ 0.7 for the forward direction.

All unique solutions in (23) are greater than or equal to 4 for any *τ* ≥ 0, as shown in Fig. 1(a).

### Backward phase transition

Set a rotating frame with the average phase of the system,

24 $$\varphi (t)=\varphi (0)+\langle \omega \rangle t$$

Here 〈*ω*〉 is the average frequency of the oscillators. For an even symmetric distribution of *g*(*ω*), 〈*ω*〉 = 0. Hence, *ϕ*(*t*) = *ϕ*(0), *ϕ*(*t* − *τ*) = *ϕ*(0). With Δ*θ*_*i*_(*t*) = *θ*_*i*_(*t*) − *ϕ*(0), then Δ*θ*_*i*_(*t*) = *θ*_*i*_(*t*) − *ϕ*(*t* − *τ*), the mean field equation (5) can be transferred into:

25 $${\rm{\Delta }}{\dot{\theta }}_{i}={\omega }_{i}-\kappa r|{\omega }_{i}|\,\sin ({\rm{\Delta }}{\theta }_{i})$$

In the coherent state, all the oscillators are phase locked. So ${\rm{\Delta }}{\dot{\theta }}_{i}=0$.

26 $${\rm{\Delta }}{\theta }_{i}=\{\begin{array}{ll}\arcsin (\frac{1}{\kappa r}) & {\omega }_{i} > 0\\ \arcsin (\,-\,\frac{1}{\kappa r}) & {\omega }_{i} < 0\end{array}$$

27 $$r=\frac{1}{N}\,\sum _{j=1}^{N}\,{e}^{i{\rm{\Delta }}{\theta }_{j}}=\frac{1}{2}({e}^{i{\rm{\Delta }}{\theta }_{i+}}+{e}^{i{\rm{\Delta }}{\theta }_{i-}})$$

Δ*θ*_*i*+_ and Δ*θ*_*i*−_ represents the two groups in the equation (26).

28 $$r=\frac{1}{2}(\cos ({\rm{\Delta }}{\theta }_{i+})+\,\cos ({\rm{\Delta }}{\theta }_{i-}))$$

As $\sin ({\rm{\Delta }}{\theta }_{i+})=\frac{1}{\kappa r}$, $\sin ({\rm{\Delta }}{\theta }_{i-})=-\,\frac{1}{\kappa r}$ and $r=\,\cos ({\rm{\Delta }}{\theta }_{i+})=\,\cos ({\rm{\Delta }}{\theta }_{i-})=\sqrt{1-{(\frac{1}{\kappa r})}^{2}}$, we have

29 $${r}^{2}=\sqrt{{r}^{2}-\frac{1}{{\kappa }^{2}}}.$$

The above equation (29) has four possible solutions of *r*(*κ*) for different *κ*, all of which are complex for *κ* < 2, two of which are positive for *κ* ≥ 2, and only one of which increases for increasing *κ*. Thus, the equation for a viable solution is: $r(\kappa )=\sqrt{\frac{\kappa +\sqrt{(\kappa -2)\,(\kappa +2)}}{2\kappa }}$, for which the first solution with zero imaginary part occurs at *κ* = 2. Hence, the backward critical value of *κ* becomes *K*_*b*_ = 2 for any even, symmetric distribution function *g*(*ω*) (Fig. 1(b)). The inset in Fig. 1(b) shows the real (blue line) and imaginary (red dashed line) parts of *r*(*κ*) at different *κ*: the imaginary part becomes zero at *κ* = 2 and remains so for increasing *κ* and, at *κ* = 2, *r*(*κ*) ≈ 0.7 (predicted value of *r* at the backward transition point).

### Numerical results and extensions

**Figure 2:**
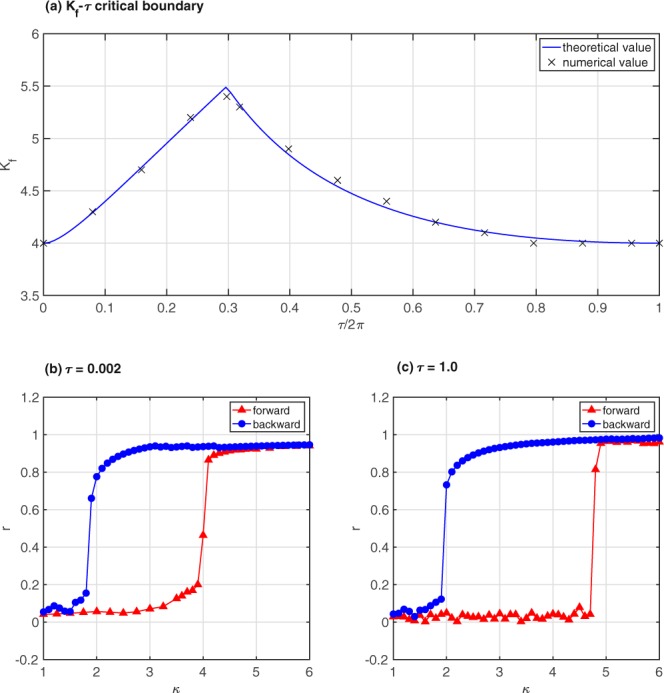
(**a**) Numerical values (x) overlaid on the theoretically predicted critical values for the forward transition (*K*_*f*_ − *τ* boundary), (**b**) *r* versus *κ* at *τ*/2*π* = 3.2 × 10^−4^, and (**c**) *r* versus *k* at *τ*/2*π* = 0.16. These numerical results are based on the RK4-integration scheme with step-size = 0.001 to solve the ordinary differential equations for 1000 coupled oscillators with incoherent initial conditions for the forward direction.

**Figure 3:**
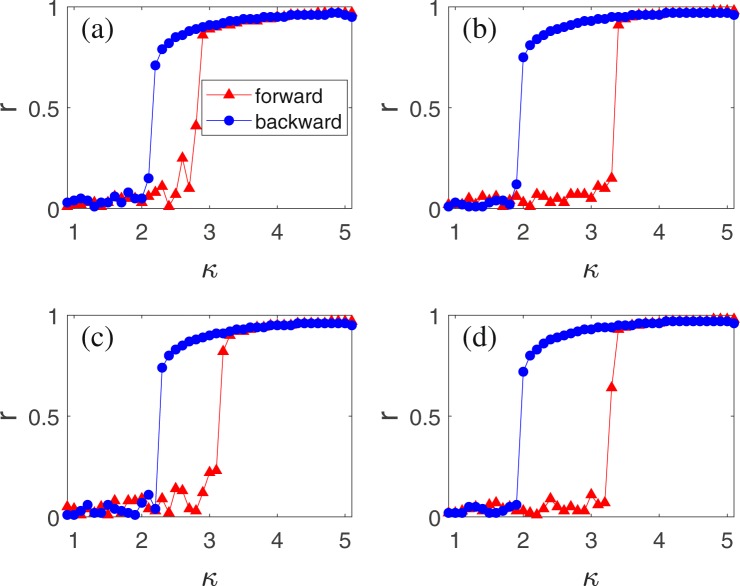
Numerical simulations on the case of random Erdos-Renyi (ER) networks with size *N* = 10^3^, where the “triangles” and “circles” represent the forward and backward processes, respectively. (**a**–**d**) Show the dependence of *r* on *κ* for different average degrees 〈*k*〉 and time delay *τ*, respectively, with (**a**) 〈*k*〉 = 10 and *τ*/2*π* = 3.2 × 10^−4^; (**b**) 〈*k*〉 = 100 and *τ*/2*π* = 3.2 × 10^−4^; (**c**) 〈*k*〉 = 10 and *τ*/2*π* = 0.16; and (**d**) 〈*k*〉 = 100 and *τ*/2*π* = 0.16.

**Figure 4:**
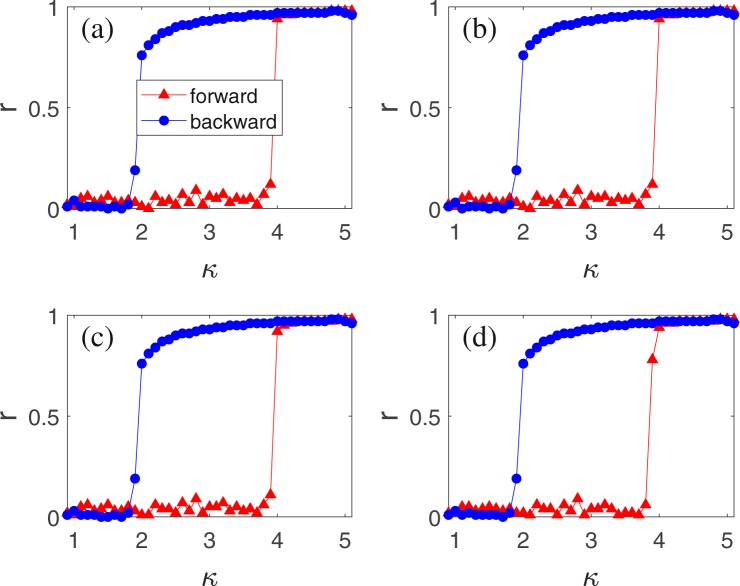
Numerical simulations on the case of fully connected networks with size *N* = 10^3^, where the “triangles” and “circles” represent the forward and backward processes, respectively. (**a**–**d**) Show the dependence of *r* on *κ* for 〈*τ*〉/2*π* = 0.16 and different *σ*, respectively, with (**a**) *σ* = 0.001; (**b**) *σ* = 0.01; (**c**) *σ* = 0.1; and (**d**) *σ* = 1.0.

As shown in Fig. 2, we numerically verify the above analytical results in a case of frequency-weighted, fixed time-delayed all-to-all coupling. We show that these findings from all-to-all, fixed time-delayed networks hold also true for the cases of sparsely connected networks (Fig. 3(a–d)) and with distributed time-delayed couplings (Fig. 4(a–d)). We now turn to extend the above results of fully connected networks to a more realistic case of sparsely connected networks. For that, we consider the random Erdos-Renyi (ER) network as an example. For an uncorrelated network, we follow refs^16,30^ to rewrite Eq. (1) as

30 $$\begin{array}{rcl}\dot{\theta }(t) & = & \omega +\kappa |\omega |\,\int \,dk^{\prime} \,\int \,d\theta ^{\prime} \tfrac{k^{\prime} P(k^{\prime} )}{\langle k\rangle }\rho (k^{\prime} ;\theta ^{\prime} ,t)\,\sin (\theta ^{\prime} (t-\tau )-\theta (t))\\  &  & -\,\kappa |\omega |h(t)\end{array}$$

where *P*(*k*), 〈*k*〉, *ρ*(*k*; *θ*, *t*) represent the degree distribution, average degree, and density of the nodes with phase *θ* at time *t* for a given degree *k*, respectively, and the term *h*(*t*) takes into account time fluctuations and is given by $h={\rm{Im}}\{{e}^{-i\theta }\,{\sum }_{j=1}^{N}\,{A}_{ij}({\langle {e}^{i{\theta }_{j}(t-\tau )}\rangle }_{t}-{e}^{i{\theta }_{j}(t-\tau )})\}$, where “Im” stands for the imaginary part. In the thermodynamic limit, the term *h*(*t*) can be neglected when the average degree 〈*k*〉 is large enough^30^. Consequently, Eq. (2) can be rewritten as

31 $$r{e}^{i\varphi (t-\tau )}=\frac{1}{\langle k\rangle }\,\int \,dk\,\int \,d\theta kP(k)\rho (k;\theta ,t){e}^{i\theta (t-\tau )}$$

which gives $r\,\sin (\varphi (t-\tau )-\theta )=\frac{1}{\langle k\rangle }\,\int \,dk\,\int \,d\theta ^{\prime} kP(k)\rho (k;\theta ^{\prime} ,t)\,\sin (\theta ^{\prime} (t-\tau )-\theta )$. Neglecting the fluctuation *h*(*t*) and substituting Eq. (31) into Eq. (30), we obtain

32 $$\dot{\theta }(t)=\omega +\kappa |\omega |r\,\sin (\varphi (t-\tau )-\theta )$$

This is exactly Eq. (5). Therefore, the results obtained from Eq. (5) should also work for the case of non-fully connected networks, provided that the term *h*(*t*) in Eq. (30) can be neglected. In numerical simulations, the ER networks with a larger average degree 〈*k*〉 will satisfy the mean-field approximation (30–32) better. Figure 3(a–d) show the dependence of *r* on *κ* for different average degrees 〈*k*〉 and time delay *τ*, respectively, with (a) 〈*k*〉 = 10 and *τ*/2*π* = 3.2 × 10^−4^; (b) 〈*k*〉 = 100 and *τ*/2*π* = 3.2 × 10^−4^; (c) 〈*k*〉 = 10 and *τ*/2*π* = 0.16; and (d) 〈*k*〉 = 100 and *τ*/2*π* = 0.16. Comparing the case of 〈*k*〉 = 100 in Fig. 3(b,d) with that of 〈*k*〉 = 10 in Fig. 3(a,c), we see that the former has a larger loop than the latter. Then, if we compare the case of 〈*k*〉 = 100 in Fig. 3(b,d) with that of fully connected network (〈*k*〉 = 999) in Fig. 2(b,c), we find that latter has a larger loop than the former. Thus, the size of loop will monotonously decrease with the decrease of 〈*k*〉. On the other hand, comparing the case of *τ*/2*π* = 3.2 × 10^−4^ in Fig. 3(a,b) with that of *τ*/2*π* = 0.16 in Fig. 3(c,d), we see that their differences are not significant, indicating that the explosive synchronization is robust to the time delay *τ*. In sum, Fig. 3(a–d) tells us that for different 〈*k*〉 and *τ*, there is always a hysteresis loop and the backward *K*_*b*_ is always close to *κ* = 2, confirming the theoretical extension of Eq. (30).

We now extend the findings to the case of non-uniform delays. For this purpose, we let each node have a different *τ*_*i*_ and let each *τ*_*i*_ be taken from a random uniform distribution with the average 〈*τ*〉/2*π* = 0.16 and standard deviation *σ*. To focus on the effect of distributed *τ*_*i*_, we take the fully connected network as an example. Figure 4(a–d) show the dependence of *r* on *κ* for different *σ*, respectively, with (a) *σ* = 0.001; (b) *σ* = 0.01; (c) *σ* = 0.1; and (d) *σ* = 1.0. We find that the backward *K*_*b*_ is always close to *κ* = 2 and the forward *K*_*f*_ is only slightly different for different *σ*, indicating that the explosive synchronization is robust to the distribution of time delay.

## Discussion and Conclusions

Here, we have generalized the Kuramoto model to include fully connected, time-delayed, frequency-weighted coupling and analytically derived the exact formulas for critical transitions to or out of explosive (abrupt, first-order like) phase synchronization and extended these results to sparsely connected networks and distributed time-delays.

We used |*ω*|-weighted coupling previously used in a non-delayed system^17^. This scheme is one of the ways to obtain and maintain explosive transitions to or out of synchronization. The frequency-based weighting scheme is relevant to many functionally organized and spatially distributed oscillator systems, some relevant examples of which include power grid networks^12,26^, social communication networks^27,28^ and brain neuronal oscillatory networks^3,29^. In the example of power grid network, a network consists of Kuramoto oscillators, where the weighted coupling coefficient between two oscillators is related to their own natural frequencies^26,27^. In the example of communication networks, an extrovert contacts his or her neighbors more frequently than an introvert. If we define the contact between two individuals as a kind of coupling and the frequency of contacts as coupling strength, the coupling strength becomes correlated with the characteristics of individuals, i.e., a kind of natural frequency of human interactions^27^.

In addition to the frequency-weighting scheme, there are other documented ways to induce and maintain explosive synchronization, which are: (i) uniform frequency distribution in all-to-all network topology^7,8,14^, (ii) time-delayed coupling^20^, (iii) order parameter-dependent coupling^13^, (iv) scale-free network topology and correlation between intrinsic frequencies and node degre^15^, and (v) coupling based on weighting procedure with network link frequency mismatch and link betweeness^31^. Taken these cases together, it leads to a general notion that additional constraints or ‘inertia’ in the system, such as avoiding close frequencies, having time-dependence in parameters or network structure - dynamics correlation, can equally induce or enhance explosive synchronization.

In sum, we generalize the Kuramoto model of globally coupled phase oscillators with time-delay and oscillation frequency-modulated coupling considering its relevance to adaptive physical, biological or technoligcal oscillators. We have analytically and numerically studied the stability of first-order synchronization in this generalized Kuramoto model. We have found the exact formulas for the critical coupling strengths at different time delays in both the increasing (forward) and decreasing (backward) directions of coupling strengths. We find that time-delay does not change the transition in the backward direction but can shift the transition for the forward direction. These results, consistent across sparsely connected networks and networks with distributed time delays, provide useful insights into our understanding of dynamical mechanisms leading to explosive synchronization in presence of often unavoidable time delays in realistic spatially distributed and functionally organized systems. We envision that our theoretical work may encourage future research on abrupt collective synchronization in models of spatially distributed and functionally organized real systems that can be mapped or reduced onto the Kuramoto model, such as Josehpon juctions^4^, cortical neurons^32^ and many more^19^.

## References

[1] Varela F, Lachaux J-P, Rodriguez E, Martinerie J (2001). The Brainweb: Phase Synchronization and Large-Scale Integration. Nat. Rev. Neuro..

[2] Engel AK, Fries P, Singer W (2001). Dynamic predictions: oscillations and synchrony in top-down processing. Nat. Rev. Neuro..

[3] Buzsaki G, Draguhn A (2004). Neuronal Oscillations in Cortical Networks. Science.

[4] Wiesenfeld K, Colet P, Strogatz SH (1996). Synchronization transitions in a disordered Josephson series array. Phys. Rev. Lett..

[5] Cawthorne AB (1999). Synchronized oscillations in Josephson junction arrays: The role of distributed coupling. Phys. Rev. B.

[6] Motter AE, Myers SA, Anhel M, Nishikawa T (2013). Spontaneous synchrony in power-grid networks. Nat. Phys..

[7] Kuramoto, Y. In *Proceedings of the International Symposium on Mathematical Problems in Theoretical Physics*, edited by Araki, H., Lecture Notes in Physics Vol. 39 (Springer, Berlin, 1975).

[8] *Chemical Oscillations, Waves, and Turbulence* (Springer, Berlin, 1984).

[9] Winfree AT (1967). Biological rhythms and the behavior of populations of coupled oscillator. J. Theor. Biol..

[10] Strogatz SH (2003). Sync: The emerging science of spontaneous order.

[11] Rodrigues FA, Peron TKDM, Ji P, Kurths J (2016). The Kuramoto model in complex networks. Phys. Rep..

[12] Boccaletti S (2016). Explosive transitions in complex networks’ structure and dynamics: Percolation and synchronization. Phys. Rep..

[13] Filatrella, G., Pedersen, N. F. & Wiesenfeld, K. Generalized coupling in the Kuramoto model. *Phys. Rev. E* **75**, 017201 1–4 (2007). 10.1103/PhysRevE.75.017201 17358294

[14] Pazo, D. Thermodynamic limit of the first-order phase transition in the Kuramoto model. *Phys. Rev. E* **72**, 046211 1–6 (2005). 10.1103/PhysRevE.72.046211 16383516

[15] Gomez-Gardenes, J., Gomez, S., Arenas, A. & Moreno, Y. Explosive Synchronization Transitions in Scale-Free Networks. *Phys. Rev. Lett*. **106**, 128701 1–4 (2011). 10.1103/PhysRevLett.106.128701 21517358

[16] Peron, T. K. DM. & Rodrigues, F. A. Explosive synchronization enhanced by time-delayed coupling. *Phys. Rev. E* **86**, 056108 1–5 (2012). 10.1103/PhysRevE.86.016102 23005486

[17] Zhang, X., Hu, X., Kurths, J. & Liu, Z. Explosive synchronization in a general complex network. *Phys. Rev. E* **88**, 010802 1–5 (2013). 10.1103/PhysRevE.88.010802 23944400

[18] Wang, Z., Tian, C., Dhamala, M. & Liu, Z. A small change in neuronal network topology can induce explosive synchronization transition and activity propagation in the entire network. *Sci. Rep*. **7**, 561 1–10 (2017). 10.1038/s41598-017-00697-5 PMC5428839 28373712

[19] Hu, X. *et al*. Exact solution for first-order synchronization transition in a generalized Kuramoto model. *Sci. Rep*. **4**, 7262 1–6 (2014). 10.1038/srep07262 PMC4248286 25434404

[20] Yeung M. K. Stephen, Strogatz Steven H. (1999). Time Delay in the Kuramoto Model of Coupled Oscillators. Physical Review Letters.

[21] Choi M. Y., Kim H. J., Kim D., Hong H. (2000). Synchronization in a system of globally coupled oscillators with time delay. Physical Review E.

[22] Dhamala, M., Ding, M. & Jirsa, V. K. Enhancement of Neural Synchrony by Time Delay. *Phys. Rev. Lett*. **92**, 074104 1–4 (2004). 10.1103/PhysRevLett.92.074104 14995856

[23] Adhikari Bhim Mani, Prasad Awadhesh, Dhamala Mukeshwar (2011). Time-delay-induced phase-transition to synchrony in coupled bursting neurons. Chaos: An Interdisciplinary Journal of Nonlinear Science.

[24] Adhikari BM, Sathian K, Epstein C, Lamichhane B, Dhamala M (2014). Oscillatory activity in neocortical networks during tactile discrimination near the limit of spatial acuity. NeuroImage.

[25] Adhikari, B. A., Epstein, C. & Dhamala, M. Localizing epileptic seizure onsets with Granger causality. *Phys. Rev. E* **88**, 030701 1–5(R) (2013). 10.1103/PhysRevE.88.030701 24125204

[26] Dorfler F, Bullo F (2012). Synchronization and Transient Stability in Power Networks and Nonuniform Kuramoto Oscillators. SIAM J. Control Optim.

[27] Wang, H. & Li, X. Synchronization and chimera states of frequency-weighted Kuramoto-oscillator networks. *Phys. Rev. E* **83**, 066214 1–4 (2011). 10.1103/PhysRevE.83.066214 21797468

[28] Xu, C. *et al*. Synchronization of phase oscillators with frequency-weighted coupling. *Sci. Rep*. **6**, 21926 1–9 (2016). 10.1038/srep21926 PMC4763290 26903110

[29] Bazhenov M, Timofeev I (2006). Thalamocortical oscillations. Scholarpedia.

[30] Restrepo, J. G., Ott, E. & Hunt, B. R. Onset of synchronization in large networks of coupled oscillators. *Phys. Rev. E* **71**, 036151 1–12 (2005). 10.1103/PhysRevE.71.036151 15903537

[31] Leyva, I. *et al*. Explosive synchronization in weighted complex networks. *Phys. Rev. E* **88**, 042808 1–7 (2013). 10.1103/PhysRevE.88.042808 24229226

[32] Sadilek, M. & Thurner, S. Physiologically motivated multiplex Kuramoto model describes phase diagram of cortical activity. *Sci. Rep*. **5**, 10015 1–8 (2015). 10.1038/srep10015 PMC4650820 25996547

